# SPEECH, HEARING AND LANGUAGE SCIENCES THERAPY IN BARIATRIC SURGERY OF THE ELDERLY: CASE REPORT

**DOI:** 10.1590/S0102-6720201500S100023

**Published:** 2015-12

**Authors:** Marlei Braude CANTERJI, Sibele Prates Miranda CORRÊA, Gabriel Sebastião de VARGAS, Jorge Laszlo Ruttkay PEREIRA, Simone Augusta FINARD

**Affiliations:** 1 Study Group on Obesity and Metabolic Surgery (GECOM); 2 Hospital de Clínicas of Porto Alegre), Porto Alegre, RS, Brazil

## INTRODUCTION

Normal aging has undergone substantial change due to the decrease in fatal infections and malnutrition, to the use of antibiotics, hormones and distribution of food. As a result, there was a decrease of the specific systemic physiological decline contributing to longevity^9^. Obesity is a chronic disease genetically characterized by excessive accumulation of fat tissue compared to lean tissue^4^. For obesity associated with aging, bariatric surgery is treatment with clear indications, although for elderly obese is necessary specific and careful assessment to identify the risks and what are the real benefits of the procedure^11^. It is crucial to address the maintenance of health in this age, because aging is biopsychosocial process that affects the individual as a whole, characterized by less functional efficiency, weakening of defense mechanisms in relation to environmental variation and loss of reserves^12^.

The stomatognathic system also undergoes changes with advancing age. The digestive process becomes slower requiring balanced diet and more appropriate in relation to consistencies and textures so that the elderly can chew effectively so that the absorption of nutrients occurs within the best possible^1^.

The phonoaudiology comes following bariatric patients recently due to structural or functional orofacial changes present in obese morphology, tone and/or mobility lips, tongue, jaw and mastigation^7^.

The protocol of the Study Group of Obesity and Metabolic Surgery (GECOM) provides interdisciplinary intervention between speech and nutrition therapy until six months after the operation. The objective of this paper is to present the speech therapy performed on an elderly undergoing bariatric surgery.

## CASE REPORT

Woman of 61 years, psychologist, 106.2 kg, 1.59 m, BMI 42 kg/m^2^ has history of obesity since adolescence. Was monitored by bariatric surgeon due to complaints of dyspnea and pain in the legs when walking. She was in psychological and psychiatric care for depression in use of paroxetine, lorazepam and clomipramida.

After medical evaluations, was conducted to psychological, speech therapy, nutrition and physiotherapy. Was released by the psychiatrist, cardiologist and pulmonologist for the surgical procedure, performed three months after the first consultation.

She began speech therapy two months before the operation. Three pre-operative consultations were carried out and, over six months, six post-operative care. Postoperative visits were made monthly, along with nutritional intervention.

Were collected the following information from clinical protocol: loss of 1^st^ left molar; alterations on tone of the muscles of the lips, tongue, cheeks and chin with sagging features; language with incoordination of movements for lifting, lowering and lateralization, and tremors. The patient had predominantly nasal breathing, and air outlet reported over the left nostril due to a deviated septum.

When she came to the assessment of oral functions, such as sucking, chewing and swallowing, it was observed that during the fluid intake in the cup, sipped content without lip seal and with interposition of the tongue. Had excessive contraction of orbicularis, while sipping a straw use. In the intake pasty food (yogurt), it was found that the extraction was performed with interposition of the lip seal and tongue. Already with solid food (bread type "candy clay"), held alternate bilateral mastication, with rotational movements of the jaw, although requiring liquid to facilitate the formation and swallowing cake. She complained of any choking with any type of consistency but especially with liquids. For improvement of these gagging, was instructed proper positioning during meals, as well as postural maneuvers (head in mild flexion) during swallowing. It was recommended the adequacy of the quantity of food placed in the fork or spoon to be taken into the mouth, straw use to avoid large amounts of liquids to be ingested and facilitating maneuvers for adequate swallowing, saliva and food with different consistencies, volume, temperature, for the remission of gagging, and performed gustative stimulation^8^.

In the second call, she reported improvement of gagging and, as the years to adjust the sensitivity, said it was "very good nothing to do with his mouth than eat." Reported some difficulty with the use of straw as it limited the amount she wanted to consume. On the other hand, she was concerned referring drank much more before the operation. After the procedure, she reported that ingested liquid quickly and felt sense of esophageal stopping, saying it should maintain attention during meals.

During the six months post-surgery, the introduction of food consistency occurred efficiently. There were two episodes of vomiting due to food intake not yet released by the professionals. After resuming again to the recommendations, the patient arrived at the end of the treatment by eating food of any consistency and texture. After six months, she lost 29.2 kg reaching 77 kg and BMI 30.4 kg/m^2^ and three months after surgery, there was improvement in depression and performed regular exercise.

## DISCUSSION

In a literature review, studies were presented that describe the close relationship between overweight and tendency to social isolation, stress, depression and worsening of functional capacity of the obese^3^. In contrast, it was found that grade III obese individuals when operated regained their self-esteem and quality of life for subsequent maintenance of weight below the levels regarded as morbid obesity^14^.

In the case of orofacial functions, oral motor disorder most referenced by the elderly is chewing change because, although indentations, the elderly do not prepare food for swallowing with the same young adult efficiency. The increase in oral transit time of the bolus, preceding the pharyngeal phase of swallowing is common in elderly^10^. It was also observed this aspect in this case. Furthermore, require significantly larger number of swallows for cleaning the pharynx^13^ and decrease in oropharyngeal sensitivity changes, so favoring microaspirations^2^, which was observed in the studied patient.

The individual seeking for operation, as an alternative to improving his/her quality of life, should be reminded the existence of oral myofunctional changes that happen in the course of life associated with aging. This leads to make a conscious and functional way what was happening automatically and inappropriately. New posture is guided to the intake in order to facilitate the reintroduction of postoperatively food. Stand out from the information provided about the masticatory process and the changes that occur in it, throughout life, has great importance for the prevention of disorders^5^. The myofunctional speech therapy, in order to raise awareness and re-enable the individual performance of speech functions, breathing, sucking, chewing and swallowing, implies life modifications^6^.

This process increases the ability, which was confirmed considering the good performance of orofacial functions accompanied the elderly.

Thus, preventive action and/or rehabilitative aims to provide well-being, active permanence of the elderly in their social environment and satisfaction with life also to bariatric patients^5^.

You need to consider that the individual search operation, better quality physical, mental and social, also covers aesthetic and functional aspects related to food. This is critical at later ages because the permanence of social interactions is closely related to successful aging, also observed as the patient described in this paper, because before the operation had limitations, reducing social activities, also by virtue of comorbidities. Importantly it has been found relation between overweight and tendency to social isolation, stress, depression and worsening of functional capacity^14^.

Taking into account the influence of speech in bariatric surgery in orofacial characteristics related to aging, are more relevant, given that there will be major change in diet after surgery. The speech therapists will collaborate with patient undergoing gastroplasty on eating foods with different consistencies and textures, which can contribute to the prevention of health risks and better quality of life^6^.
